# Biomechanical mechanism of reduced aspiration by the Passy-Muir valve in tracheostomized patients following acquired brain injury: Evidences from subglottic pressure

**DOI:** 10.3389/fnins.2022.1004013

**Published:** 2022-10-31

**Authors:** Xiaoxiao Han, Qiuping Ye, Zhanao Meng, Dongmei Pan, Xiaomei Wei, Hongmei Wen, Zulin Dou

**Affiliations:** ^1^Department of Rehabilitation Medicine, The Third Affiliated Hospital of Sun Yat-sen University, Guangzhou, China; ^2^Department of Radiology, The Third Affiliated Hospital of Sun Yat-sen University, Guangzhou, China; ^3^School of Mechanical and Automotive Engineering, South China University of Technology, Guangzhou, China

**Keywords:** aspiration, tracheostomy, acquired brain injury, Passy-Muir Tracheostomy and Ventilator Swallowing and Speaking Valve (PMV), swallowing biomechanics, subglottic pressure

## Abstract

**Objective:**

Aspiration is a common complication after tracheostomy in patients with acquired brain injury (ABI), resulting from impaired swallowing function, and which may lead to aspiration pneumonia. The Passy-Muir Tracheostomy and Ventilator Swallowing and Speaking Valve (PMV) has been used to enable voice and reduce aspiration; however, its mechanism is unclear. This study aimed to investigate the mechanisms underlying the beneficial effects of PMV intervention on the prevention of aspiration.

**Methods:**

A randomized, single-blinded, controlled study was designed in which 20 tracheostomized patients with aspiration following ABI were recruited and randomized into the PMV intervention and non-PMV intervention groups. Before and after the intervention, swallowing biomechanical characteristics were examined using video fluoroscopic swallowing study (VFSS) and high-resolution manometry (HRM). A three-dimensional (3D) upper airway anatomical reconstruction was made based on computed tomography scan data, followed by computational fluid dynamics (CFD) simulation analysis to detect subglottic pressure.

**Results:**

The results showed that compared with the non-PMV intervention group, the velopharynx maximal pressure (VP-Max) and upper esophageal sphincter relaxation duration (UES-RD) increased significantly (*P* < 0.05), while the Penetration-Aspiration Scale (PAS) score decreased in the PMV intervention group (*P* < 0.05). Additionally, the subglottic pressure was successfully detected by CFD simulation analysis, and increased significantly after 2 weeks in the PMV intervention group compared to the non-PMV intervention group (*P* < 0.001), indicating that the subglottic pressure could be remodeled through PMV intervention.

**Conclusion:**

Our findings demonstrated that PMV could improve VP-Max, UES-RD, and reduce aspiration in tracheostomized patients, and the putative mechanism may involve the subglottic pressure.

**Clinical trial registration:**

[http://www.chictr.org.cn], identifier [ChiCTR1800018686].

## Introduction

Swallowing safety refers to the full protection of the respiratory tract during swallowing, thereby preventing an alimentary bolus or liquid from entering the trachea or lungs. When the airway protection function decreases during swallowing, swallowing safety is impaired and penetration or aspiration occurs (Prescott et al., 2020). Patients with aspiration are 11 times more likely to develop aspiration pneumonia compared to those without aspiration (Chiavaroli et al., 2016). The Global Tracheostomy Collaborative reported that patients who underwent tracheostomy were subject to a wide range of risks and complication that warrant efforts to address safety, including tracheostomy-related hemorrhage, accidental, and failed decannulation, prolonged length of stay, a higher mortality rate (Brenner et al., 2020). Furthermore, patients who underwent tracheostomy had a higher rate of aspiration, which increased with the duration of tracheostomy (Harris et al., 2012; Kothari et al., 2017). The endotracheal tube may be the most relevant factor responsible for pneumonia development (Coppadoro et al., 2019). Up to 87% of patients with acquired brain injury (ABI) whom received tracheostomies might suffer from aspiration, resulting in aspiration pneumonia and difficulty in extubation, placing a heavy burden on families and society (Bader and Keilmann, 2017; Enrichi et al., 2017).

Tracheostomy after ABI is associated with a series of pathological changes in the biomechanics of the upper respiratory tract, pharynx, and esophagus (Fernandez-Carmona et al., 2012). In particular, the reduction of glottic airflow after tracheostomy results in a corresponding inability to increase subglottic pressure during swallowing; as a result, normal respiratory-swallowing coordination is impaired, increasing the risk of aspiration (Fernandez-Carmona et al., 2012; Heidler, 2019b). Subglottic pressure is defined as the pressure measured when the vocal cords are adducted, and has been proved to be closely associated with swallowing function in tracheostomized patients (Petekkaya et al., 2019). As an important component of safe swallowing, the positive subglottic pressure during swallowing can build an air barrier to prevent aspiration and push the bolus into the esophagus (Gross et al., 2012). However, some studies have suggested that aspiration might be caused by ABI, rather than tracheostomy (Bader and Keilmann, 2017; Goff, 2017), which remains controversial. These findings suggest that biomechanical changes in the upper airway after tracheostomy are important factors that affect swallowing safety and may result in aspiration. This hypothesis provides the rationale for the current investigation of the Passy-Muir Tracheostomy and Ventilator Swallowing and Speaking Valve (PMV) mechanism of prevention of aspiration after tracheostomy in patients with ABI. Furthermore, a previous study indicated that long-term tracheostomy use might reduce laryngeal elevation and sensitivity of the larynx and may lead to disuse atrophy of the laryngeal musculature, which also predispose patients to an increased risk of aspiration (Li et al., 2021). Recent literature has explored the mechanism of impaired swallowing safety in patients with chronic oropharyngeal dysphagia from the perspective of the motor and sensory pathways of swallowing through repeated transcranial magnetic stimulation to detect pharyngeal motor evoked potential (pMEP) and pharyngeal sensory evoked potential. However, no direct relationship has been found between pMEP and aspiration (Cabib et al., 2020). Therefore, further evidence is needed to elucidate the underlying mechanism of swallowing safety.

The Speaking Valve (PMV), a check valve used to help tracheostomized patients with vocalization, has been demonstrated to enable these patients to speak effectively (Adam et al., 2015). It has been proved that speaking valve placement within 24 h of percutaneous tracheostomy was feasible (Martin et al., 2021). Literature has shown that PMV could also improve cough, decrease secretions, and reduce aspiration (Suiter et al., 2003). PMV has been confirmed to alleviate or eliminate the symptoms of aspiration after tracheostomy in ABI patients and significantly shorten the time of tracheal cannula insertion (Elpern et al., 2000). In fact, PMV has been widely used clinically in Europe and America to improve swallowing function and speaking and prevent aspiration (Fröhlich et al., 2017); however, studies involving PMV are scarce in China. On the other hand, studies have suggested that PMV intervention has no obvious effect on swallowing biomechanics or aspiration conditions (Prigent et al., 2012; Srinet et al., 2015), resulting in controversy surrounding PMV. As for its mechanism, researchers have considered that PMV might reconstruct the complete airway pathway, restoring physiological subglottic pressure, and upper airway fluid dynamic characteristics (Tan et al., 2017); however, there is a lack of sufficient evidence to support this.

To investigate the mechanism of PMV intervention for aspiration after tracheostomy in ABI, we focused on biomechanical changes in subglottic pressure. Based on the literature on subglottic pressure detection, we found that Gross used cricothyroid membrane puncture, which could directly measure subglottic pressure; however, this is an invasive procedure with poor patient cooperation (Gross et al., 2006). Alternatively, subglottic pressure was inferred through changes in lung volume, but this method presented with limitations related to ecological validity (Desjardins et al., 2021). Recently, a neck-surface accelerometer was developed, which uses subglottal impedance-based inverse filtering to estimate unsteady glottal airflow, which is a simple methodology, but anatomical changes were not taken into account (Ibarra et al., 2021). In recent years, computational fluid dynamics (CFD) have been used to diagnose and evaluate obstructive airway diseases (Balakrishnan and Chockalingam, 2017; Song et al., 2019). It can obtain the relevant information of a certain fluid under specific conditions and carries out the test by computer instead of test device, providing the operating platform for real simulation (Shirazawa et al., 2020). Furthermore, the viscosity of a liquid bolus at the pharynx and pharyngeal airway were predicted by CFD (Ohta et al., 2019; Shirazawa et al., 2020). Thus, CFD might be a good way to estimate subglottic pressure using a non-invasive method. However, no relevant studies have investigated fluid dynamic changes in the upper airway of tracheostomized patients with aspiration.

This study aimed to investigate the subglottic pressure and swallowing biomechanics of aspiration after tracheostomy in patients with ABI treated with PMV and to explore the underlying mechanism of aspiration prevention.

## Materials and methods

### Subjects

**FIGURE 1:**
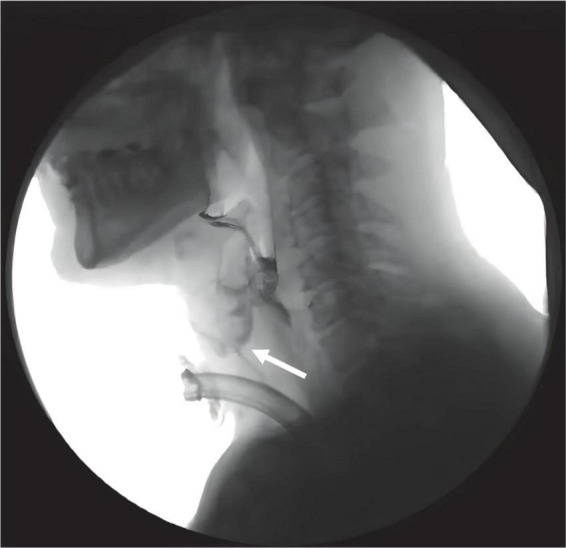
Aspiration occurred during swallowing in tracheostomized patients following ABI during VFSS examination. White arrow indicates the bolus entering the upper airway.

Tracheostomized patients with aspiration following ABI were enrolled in this study. The inclusion criteria were as follows: (1) diagnosis of ABI; (2) age between 18 and 80 years; (3) ABI occurs within 1–12 months; (4) use of a tracheostomy tube; (5) non-ventilated; (6) without supplementary oxygen requirements; (7) clinical symptoms of aspiration [Penetration-Aspiration Scale (PAS) score ≥ 5, Figure 1]; and (8) no medical history of previous dysphagia. The exclusion criteria were as follows: (1) unstable vital signs; (2) severe cognitive impairment; (3) inability to achieve an upright sitting position; (4) medical history of seizures; (5) allergy to iohexol injections; (6) pregnant or nursing women; and (7) any neuropsychiatric comorbidity or affective disorder that may influence the test outcomes. Written informed consent was obtained from all the participants or their relatives prior to inclusion.

**FIGURE 2:**
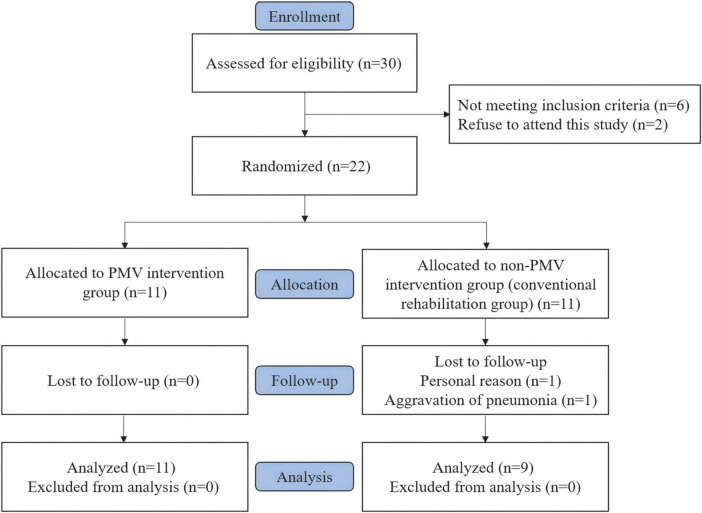
Flow diagram of the inclusion process of the study.

Thirty tracheostomized patients with aspiration following ABI were evaluated for eligibility, of which 22 patients who met the inclusion criteria were included and equally randomized into two groups. Two patients in the non-PMV intervention group discontinued the treatment. Therefore, 11 patients in the PMV intervention group and 9 patients in the non-PMV intervention group completed the study. Figure 2 shows a flow diagram of the inclusion process of the study.

### Study design

This study was designed as a randomized, single-blinded, controlled trial. All data were obtained from the Third Affiliated Hospital of Sun Yat-sen University. The patients were divided into two groups by a random allocation sequence generated by a computer random number generator. All assessments were conducted in a single (assessor) blind manner.

The PMV intervention group received a PMV intervention combined with conventional treatment. Oral cleaning and sputum suction were performed before the PMV intervention. The patient remained in a semi-recumbent position and the PMV was secured to the tracheostomy tube. Patients in this group also received conventional therapies for 30 min (once daily, 5 days per week). These therapies included effortful swallowing, Mendelssohn’s maneuver, supraglottic swallow, oropharyngeal muscle strengthening exercises, and postural compensation for head rotation. Patients in the non-PMV intervention group received the same conventional therapies as those in the PMV intervention group.

### Data collection

#### High-resolution manometry procedure

A high-resolution solid-state pressure measurement system was used to examine the pharyngeal pressure (Sierra Scientific Instruments, Los Angeles, CA, USA). The device used the proprietary pressure transduction technology (TactArray) which allowed each of the 36 pressure sensing elements to detect pressure over a length of 2.5 mm in each of the 12 circumferentially dispersed sectors. The sector pressures were then averaged to obtain the mean pressure measurement, making each of the 36 sensors a circumferential pressure detector. The change in pressure was directly displayed as the change in the electrical signal on the sensor. All pressure measurements were expressed in terms of atmospheric pressure.

**FIGURE 3:**
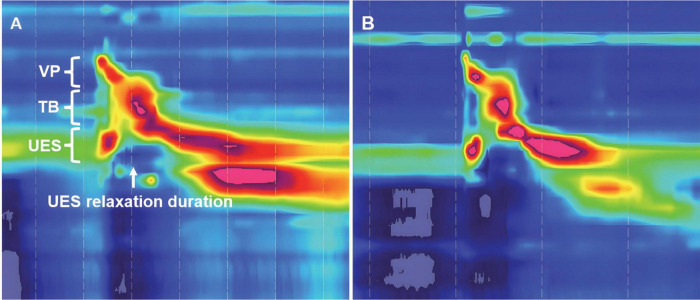
Time-space chart of high-resolution manometry in a tracheostomized patient with aspiration following ABI. **(A)** At baseline before Passy-Muir Tracheostomy & Ventilator Swallowing and Speaking Valve (PMV) intervention, the pressure detected region of velopharynx (VP), tongue base (TB), and upper esophageal sphincter (UES) are shown, white arrow indicates the UES relaxation duration; **(B)** 2 weeks after PMV intervention in the same patient.

Before examination, oral cleaning, sputum suction, and nasogastric feeding tube extraction were performed. All subjects then underwent transnasal placement of the manometric assembly in a natural sitting position with their head in a neutral position. Real-time pressure imaging during catheter intubation enables accurate placement. The manometric catheter was positioned to record from the velopharynx (VP) to the upper esophageal sphincter (UES). The catheter was fixed in place by taping it to the nostril. After a quiet resting adaptation period of more than 10 min, each subject was instructed to swallow 5 mL iohexol injection for a total of three swallows. Pressure and timing data were analyzed using ManoView analysis software (Sierra Scientific Instruments, Los Angeles, CA, USA). Three regions of interest were identified: the VP, tongue base (TB), and UES (Figure 3). VP was defined as the zone of swallow-related pressure change proximal to the region of continuous nasal nostril quiescence, extending 2 cm distally. Anatomically, VP is defined as the soft palate and posterior pharynx (McCulloch et al., 2010; Park et al., 2017). TB was defined as the zone of swallow-related pressure change with a high-pressure area midway between the VP and UES, with its center at the maximal pressure point and extending 2 cm proximal and distal to that point (McCulloch et al., 2010; Park et al., 2017). The UES region was defined as the midpoint of stable high pressure just distal to the baseline low esophageal pressure zone (McCulloch et al., 2010; Park et al., 2017). The parameters included VP maximal pressure (VP-Max), TB maximal pressure (TB-Max), UES residual pressure (UES-RP), and UES relaxation duration (UES-RD).

#### Video fluoroscopic swallow study procedure

The subjects were placed in a neutral sitting position under the guidance of a C-arm remote control twin-perspective gastrointestinal X-ray machine (Toshiba DBA-300, Toshiba Ltd., Co., Japan). Each subject was instructed to swallow 5 mL iohexol injection for a total of three swallows. The severity of laryngeal penetration during swallowing was measured using the 8-point PAS. A digital acquisition and analysis system for video fluoroscopy was used to quantitatively analyze the video fluoroscopic swallow study (VFSS) video. The video was recorded during the VFSS and played frame by frame, capturing the target frame, which is when the hyoid was located at the lowest/highest point and the laryngeal vestibule was open/closed during swallowing. Illustrations were measured and calculated using Image J software (National Institute of Mental Health, Bethesda, MD). These parameters included laryngeal vestibule closure time (LVC), anterior hyoid displacement (AHD), upper hyoid displacement (UHD), and PAS score. The LVC was the total duration the laryngeal vestibule remains closed during the pharyngeal stage of swallowing. The AHD was the displacement of the hyoid bone in an anterior direction. The UHD was the displacement of the hyoid bone in an upward direction.

#### Reconstruction of the upper airway model

A computed tomography (CT) scanner (Aquilion ONE; Toshiba Medical Systems Corp., Tokyo, Japan) was used. The full sequence was performed in approximately 8.9 s by the same operator. The tube voltage/current ratio was 120 kV/60 mA. Cross-sectional CT images of the upper airway with a 0.5-mm thickness were obtained in the coronal, sagittal, and axial planes. Subjects were placed in the supine position without PMV during CT examination. And subjects did not swallow anything during CT examination. The dataset was read and analyzed using Mimics software (version 20.0; The Materialize Group, Leuven, Belgium) to construct the 3D model. An appropriate smoothing algorithm was used to transform the 3D model into a smooth model without loss of patient-specific characteristics of the shape of the upper airway. Subsequently, stereolithography files of the 3D models were imported into ANSYS 15.0-Meshing (Canonsburg, PA, USA) for model repair and mesh generation.

#### Computational fluid dynamics

After mesh generation, the mesh file was imported into ANSYS FLUENT 15.0, and the internal flow of the upper respiratory tract was simulated. In the simulation process, the upper airway was regarded as a rigid cavity, and the fluid flowed in an incompressible manner with constant viscosity.

**FIGURE 4:**
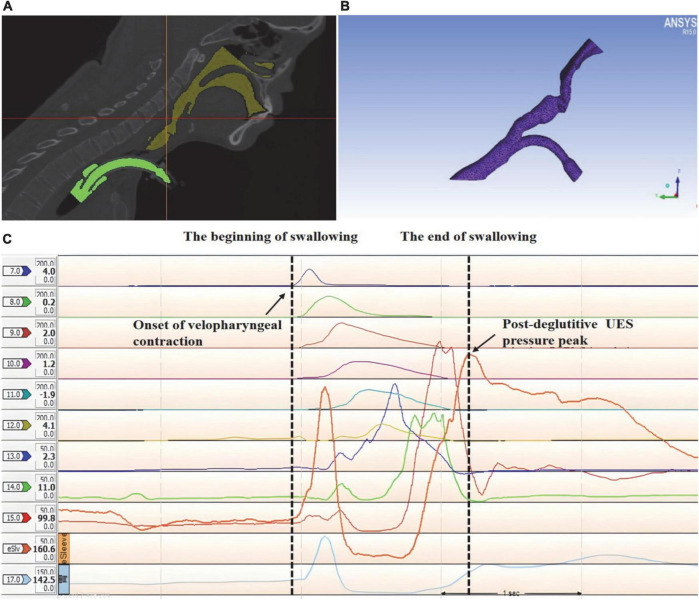
3D reconstruction and mesh generating of the upper airway anatomical structure in a tracheostomized patient with aspiration following acquired brain injury (ABI) based on high-resolution manometry (HRM). **(A)** 3D reconstruction of the upper airway anatomical structure, yellow: upper respiratory tract; green: tracheal tube; **(B)** mesh generating of the upper airway 3D geometry; **(C)** the duration of swallowing was analyzed using HRM.

Two different periods, a respiratory and swallowing period, were considered in the simulation. In this study, the respiratory cycle duration was T = 3 s. The duration of swallowing was defined as the time between the onset of velopharyngeal contraction and post-deglutitive UES pressure peak (Figure 4). The pressure inlet was defined as the boundary condition for palatopharyngeal entrance. The pharyngeal pressure over time before swallowing, measured using high-resolution manometry (HRM), was used as the pressure value. During the respiratory period, the outlet of the subglottic cavity was defined as the velocity outlet, the value of which is defined as the respiration volume. During the swallowing process, the outlet of the subglottic cavity is at the pressure exit. The k-ϵ Standard Reynolds model was used for evaluating the turbulent flow within the upper respiratory tract. The subglottic pressure generated during swallowing varies over time. The mean subglottic pressure during swallowing was calculated using calculus.

Two different group simulations were conducted to investigate the effects of the PMV intervention on the subglottic pressure. Therefore, for the non-PMV intervention group, the pressure at the PMV inlet was defined as the air pressure. For the PMV intervention group, the inlet of the PMV was defined as the wall boundary condition during exhalation, whereas the inlet of the PMV was defined as the pressure boundary condition during inhalation.

#### Outcome measurements

All participants were evaluated prior to and 2 weeks after treatment. Patients were examined with the tracheal cuff inflated prior to and 2 weeks after treatment in the HRM and VFSS procedure in the non-PMV intervention group. Patients were examined with the tracheal cuff inflated prior to treatment in the HRM and VFSS procedure in the PMV intervention group. Patients were examined with PMV after treatment in the HRM and VFSS procedure in the PMV intervention group and the tracheal cuff was deflated during the examinations. The primary outcome was the PAS score, whereas the secondary outcomes included subglottic pressure, VP-Max, TB-Max, UES-RP, UES-RD, LVC, AHD, and UHD.

#### Statistical analysis

The sample size was calculated such that at least nine individuals from each group had to be included in this study for 80% power with a 5% type I error level to detect a significant difference in PAS score (Park et al., 2013).

All statistical analyses were performed using SPSS 23.0 (SPSS Inc., Chicago, IL, USA). Data are presented as mean ± standard deviation (SD). Categorical variables are presented as frequencies. Normally distributed data were determined using the Shapiro-Wilk test. Homogeneity of variance was measured using the Levene’s test. In terms of clinical characteristics, sex, brain injury etiology, lesion location, lesion side, and presence of pulmonary infection were expressed as the number of participants and analyzed using Fisher’s exact test. Continuous variables of the baseline characteristics between the groups were expressed as mean ± SD and were analyzed using the two-tailed two independent samples *t*-test or Wilcoxon signed-rank test. The two-tailed paired samples *t*-test or Wilcoxon signed-rank test was used for changes in parameters prior to and after treatment in the PMV and non-PMV intervention groups. These tests were also used to compare the differences between the two groups before and after treatment (post-pre treatment). The level of statistical significance was set at *P* < 0.05.

## Results

### Effect of Passy-Muir tracheostomy and ventilator swallowing and speaking valve intervention on the swallowing biomechanics of tracheostomized patients with aspiration following acquired brain injury

The characteristics of patients in the two groups are displayed in Table 1. The biomechanical characteristics of swallowing in the two groups at baseline are shown in Table 2. There were no significant differences between the two groups at baseline in terms of age, sex, body mass index, brain injury etiology, lesion location, lesion side, National Institute of Health Stroke Scale score (for stroke), Functional Independence Assessment score, Functional Oral Intake Scale score, time from disease onset, duration of tracheal intubation, presence of pulmonary infection, VP-Max, TB-Max, UES-RP, UES-RD, LVC, AHD, UHD or PAS score.

**TABLE 1:** Clinical characteristics in the PMV and non-PMV intervention groups.

	PMV intervention group (*N* = 11)	Non-PMV intervention group (*N* = 9)	*P*-value
Age (years)	57.13 ± 10.51	64.21 ± 5.19	0.222
Sex, male, *n* (%)	7 (63.64%)	4 (44.44%)	0.653
BMI (kg/m^2^)	20.14 ± 2.44	18.00 ± 2.09	0.120
Brain injury etiology, *n* (%)			0.835
Stroke	7 (63.64%)	5 (55.56%)	
Brain tumor	2 (18.18%)	1 (11.11%)	
Traumatic brain injury	2 (18.18%)	3 (33.33%)	
Lesion location, *n* (%)			0.642
Supratentorial	3 (27.27%)	4 (44.44%)	
Infratentorial	8 (72.73%)	5 (55.56%)	
Lesion side, *n* (%)			0.390
Left	3 (27.27%)	4 (44.44%)	
Right	6 (54.55%)	2 (22.22%)	
Both	2 (18.18%)	3 (33.33%)	
NIHSS (for stroke)	6.72 ± 3.07	7.56 ± 2.24	0.509
FIM	62.18 ± 9.17	63.89 ± 5.49	0.630
FOIS	1.55 ± 0.52	1.33 ± 0.50	0.355
Time from disease onset (months)	2.68 ± 0.90	2.81 ± 0.96	0.760
Duration of tracheal intubation (months)	2.62 ± 0.88	2.76 ± 0.95	0.748
Pulmonary infection, *n* (%)	9 (81.82%)	8 (88.89%)	>0.999

BMI, body mass index; NIHSS, National Institute of Health Stroke Scale; FIM, Functional Independence Assessment; FOIS, Functional Oral Intake Scale. Sex, brain injury etiology, lesion location, lesion side, and presence of pulmonary infection were expressed as the number of participants and analyzed with the Fisher’s exact test. Other characteristics were expressed as the mean ± standard deviation and analyzed with the two-independent sample *t*-test or Wilcoxon signed-rank test.

**TABLE 2:** Swallowing biomechanical characteristics and subglottic pressure in PMV and non-PMV intervention groups at baseline.

	PMV intervention group (*N* = 11)	Non-PMV intervention group (*N* = 9)	*P*-value
VP-Max	95.01 ± 53.91	104.86 ± 77.11	0.790
TB-Max	117.38 ± 56.45	103.90 ± 71.98	0.644
UES-RP	17.23 ± 15.35	14.41 ± 11.48	>0.999
UES-RD	444.91 ± 127.52	472.67 ± 57.01	0.554
LVC	771.18 ± 237.66	783.89 ± 210.34	0.902
AHD	3.36 ± 1.81	3.49 ± 1.23	0.422
UHD	12.39 ± 4.89	10.29 ± 3.75	0.305
PAS score	6.82 ± 1.47	7.44 ± 0.53	0.448
Subglottic pressure	0.53 ± 0.10	0.60 ± 0.06	0.243

Data were expressed as the mean ± SD and analyzed with the two-independent sample *t*-test or Wilcoxon signed rank test.

To observe the effect of the PMV intervention, the swallowing biomechanical parameters were compared between the PMV intervention and non-PMV intervention groups. The representation figure of the VFSS is shown in Figure 1. Swallowing biomechanics were measured using HRM prior to and 2 weeks after PMV intervention (Figure 3).

**FIGURE 5:**
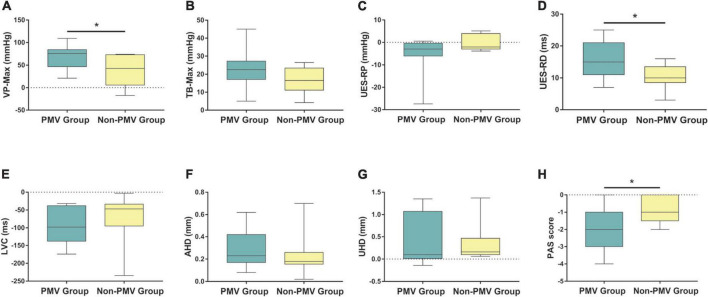
Comparisons of the differences of post-pre treatment between two groups in swallowing biomechanical characteristics and subglottic pressure. **(A–H)** Velopharynx maximal pressure (VP-Max), tongue base maximal pressure (TB-Max), upper esophageal sphincter residual pressure (UES-RP), UES relaxation duration (UES-RD), laryngeal vestibule closure time (LVC), anterior hyoid displacement (AHD), upper hyoid displacement (UHD), and Penetration-Aspiration Scale (PAS) score. Data were expressed as the mean ± standard deviation and analyzed with the two-independent sample *t*-test or Wilcoxon signed rank test. *P < 0.05.

The results demonstrated that VP-Max and UES-RD increased and PAS scores decreased significantly in the PMV intervention group compared to the non-PMV intervention group (*P* < 0.05). However, there were no statistical differences in TB-Max, UES-RP, LVC, AHD, or UHD (*P* > 0.05) (Figures 5A–H). These results indicate that PMV can improve VP-Max, UES-RD, and aspiration.

### Three-dimensional reconstruction of the upper airway anatomical structure of patients and analysis of subglottic pressure by computational fluid dynamics

**FIGURE 6:**
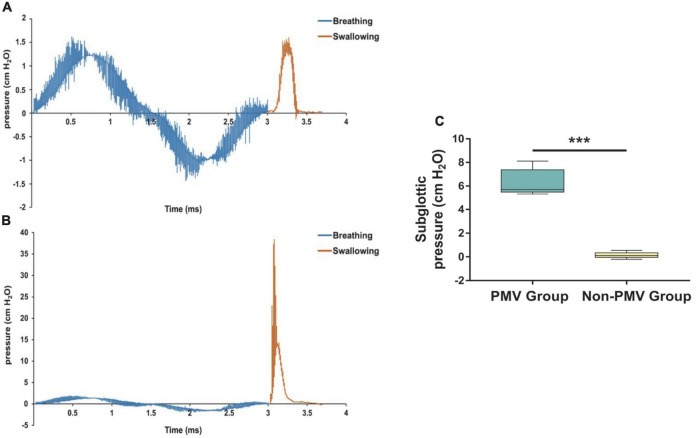
Comparisons of the differences prior to and after treatment of subglottic pressure between the PMV intervention and non-PMV intervention groups. **(A)** Sample of the subglottic pressure generated during swallowing varied with time in tracheostomized patient with aspiration following acquired brain injury (ABI) at baseline before PMV intervention; **(B)** 2 weeks after PMV intervention in the same patient; **(C)** comparison of the differences prior to and after treatment of subglottic pressure between two groups. Data were expressed as the mean ± standard deviation and analyzed with the Wilcoxon signed rank test. ***P < 0.001.

To investigate the subglottic pressure in tracheostomized patients with aspiration following ABI, a 3D upper airway anatomical reconstruction was made based on CT scans and HRM data (Figure 4). Subsequently, the subglottic pressure was analyzed using CFD. There was no significant difference between the two groups at baseline in subglottic pressure (Table 2). Results showed that the subglottic pressure increased from 0.53 to 6.95 cmH_2_O after PMV intervention, indicating that subglottic pressure could be remodeled through PMV intervention (Figure 6). Additionally, the subglottic pressure was higher in the PMV intervention group than in the non-PMV intervention group (*P* < 0.001) (Figure 6). These results demonstrated that the subglottic pressure was remodeled by PMV intervention.

## Discussion

In our study, the biomechanical mechanism of aspiration prevention by PMV was explored in tracheostomized patients following ABI. The results showed that, compared with the non-PMV intervention group, VP-Max, UES-RD, and subglottic pressure significantly increased, while PAS scores decreased in the PMV intervention group. We concluded that PMV intervention could improve aspiration and remodel subglottic pressure after tracheostomy in patients with ABI.

In the present study, 3D reconstruction of the upper airway anatomical structure combined with CFD analysis was used to measure the subglottic pressure, as described in a previous study (Zheng et al., 2017). Remarkably, the pharyngeal pressure measured by HRM was defined as the boundary condition of the CFD analysis. Overall, it is a non-invasive method that can calculate the subglottic pressure and is suitable for all participants. To the best of our knowledge, this is the first study to measure subglottic pressure using CFD analysis. In tracheostomy patients with aspiration, the subglottic pressure was about 0.53 cmH_2_O, while it reached 6.95 cmH_2_O after PMV intervention. These results detected by the 3D reconstruction of the upper airway anatomical model and CFD nearly reached the values of subglottic pressure that were measured by direct detection methods in previous research (Gross et al., 2006), with the values ranging from 5.5 to 9.5 cmH_2_O in healthy subjects. Lung volume also affects the subglottic pressure after tracheostomy, in which the subglottic airflow escapes from the tracheal cannula, resulting in lower subglottic pressure (Gross et al., 2003; Heidler, 2019a). Therefore, the subglottic pressure was detected with a relatively high accuracy in our study.

### Speculation on the underlying mechanism of aspiration prevention by Passy-Muir tracheostomy and ventilator swallowing and speaking valve after tracheostomy in patients with acquired brain injury

There is a highly stable and coordinated relationship between swallowing and respiration in healthy adults, in which swallowing usually occurs during the expiratory phase and further exhalation occurs after swallowing (Rattanajiajaroen and Kongpolprom, 2021). After tracheostomy, a significant and persistent expiratory airflow leak occurs, which might counteract the protective effect of expiration on the upper airway, resulting in unsafe swallowing, and possible aspiration (Eibling and Gross, 1996). The decrease in subglottic pressure after tracheostomy severely affects the normal respiratory-swallowing coordination model (Gross, 2009; Fernandez-Carmona et al., 2012; Heidler, 2019b). Previous research has shown that PMV can improve verbal communication and swallowing, especially in the swallowing-breathing coordination model (Prigent et al., 2012; Adam et al., 2015; Fröhlich et al., 2017). The presence of tracheal sleeves after tracheostomy often aggravates the occurrence of aspiration and possible mechanisms are related to the decrease in subglottic pressure during swallowing (Logemann et al., 1998). We speculated that remodeling of the subglottic pressure by PMV intervention was a key factor in preventing aspiration.

Except for subglottic pressure, our study also found that VP-Max and UES-RD improved in the PMV intervention group compared to the non-PMV intervention group. Among them, VP-Max was thought to be the most sensitive and representative parameter reflecting pharyngeal cavity pressure (Park et al., 2016), which had a significant positive predictive effect on the occurrence of subglottic aspiration. Another study also indicated that decreased palatopharyngeal systolic pressure was an important predictor of aspiration pneumonia, similar to UES-RD (Park et al., 2017). The above study demonstrated a correlation between VP-Max and aspiration; however, non-tracheostomized patients were included. The increase in VP-Max and UES-RD might be a protective factor for the prevention of aspiration, indicated by a decrease in PAS scores, which were used to assess penetration and aspiration. The improvement of VP-Max and UES-RD combined with the increase in subglottic pressure after PMV seems to have restored the normal swallowing biomechanical parameters, which might explain the mechanism of aspiration prevention and improvement.

The theory of subglottic pressure suggests that subglottic mechanoreceptors provide respiratory-related input to the swallowing central pattern generator (CPG) (Gross et al., 2006), thereby regulating swallowing. When the subglottic pressure is zero or low, it could hinder the drive of laryngeal mechanoreceptors and reduce the transmission of a bolus. This is due to the UES opening diameter, transport time, and the upper laryngeal lift being reduced, leading to physiological changes in swallowing and aspiration. Previous literature demonstrated that the brainstem CPG might be the neural network center for coordination of swallowing and respiration (Bautista et al., 2014). Furthermore, the sensitivity of the larynx is reduced after tracheostomy. Taking into account that aspirations are linked to laryngeal sensation deficits caused by neurogenic diseases (Freitag et al., 2021), we can conclude that aspiration is controlled by the sensory system to some extent.

So far, there have been various interventions to increase the subglottic pressure, including removal of the tracheal cannula, sealing the tracheal cannula, or wearing a speaking valve (Kim et al., 2015, 2017). Air insufflation has also been used to increase the subglottic pressure during swallowing, aiming to reduce the incidence of aspiration in tracheostomized patients (Clarett et al., 2014). The speech valve has been used to reduce the occurrence of aspiration in patients who underwent tracheostomy based on the principle that the speech valve opens during inspiration and closes during expiration (Tan et al., 2017). When the speech valve is closed during expiration, subglottic pressure was restored and normal swallowing could be maintained, which further decreased the occurrence of aspiration. Combined with the results of our study that the subglottic pressure increased to approximately 6.95 cmH_2_O after 2 weeks of PMV intervention, we concluded that subglottic pressure was remodeled in the PMV intervention group. These results were consistent with relevant findings in the literature (Gross et al., 2012; Mukaihara et al., 2018), which showed that the subglottic pressure ranged from approximately 8–10 cmH_2_O after PMV intervention (Harris et al., 2012). We further speculated that remodeling of subglottic pressure in the PMV intervention group might be due to preserving subglottic flow and producing breathing airflow with the help of the PMV.

However, other results of swallowing biomechanics, such as TB-Max, UES-RP, and LVC, which were closely associated with swallowing function, showed no changes after PMV intervention compared to the non-PMV intervention group. TB-Max was thought to be related to swallowing conditions, including liquid, semisolid, and dry swallowing, which might change the sequential order, duration, and magnitude of tongue pressure production (Furuya et al., 2012). Moreover, the TB pressure was also influenced by age-related changes, which are attributed to muscle weakening and morphological changes in the oropharynx (Tamine et al., 2010; Nakao et al., 2021). This is consistent with another study that considered that TB pressure was controlled by volitional modification (Park et al., 2017). Furthermore, Srinet indicated that PMV did not improve normal pharyngeal swallow biomechanics, including hyoid bone and laryngeal movements (Srinet et al., 2015), further explaining the results of our study. As for UES-RP, literature indicates that UES opening is modulated by bolus factors, such as volume and viscosity, to facilitate bolus passage (Cock et al., 2017). UES pressure, which is centrally controlled and modulated by sensory information, always appears increased in patients after brainstem stroke (Omari et al., 2015). Interestingly, UES-RD is thought to be more sensitive and reliable than UES-RP, which is influenced by neck movement and speech during swallowing (Park et al., 2017). Referring to our previous study, repeated dilatation therapy could increase the UES-RP, further promoting the excitability of affected corticomotor projections in patients with unilateral brainstem stroke (Wei et al., 2017), indicating that the UES might be controlled by the swallowing cortex rather than the periphery.

There were studies on the effects of other devices on swallowing and aspiration in tracheostomy, such as the Blom low-profile one-way tracheotomy tube speaking valve and the one-way speaking valves in-line with the ventilator. One study reported that aspiration status was unaffected by the Blom low-profile one-way tracheotomy tube speaking valve (Srinet et al., 2015). The other study reported that the use of in-line speaking valve in tracheostomized patients accompanied by improved oral intake, and the need for modification of fluids was less frequent post introduction of in-line speaking valve (Sutt et al., 2015). In the setting of the COVID-19 pandemic, cuff deflation for one-way speaking valve use or capping is presumed to increase aerosolization of viral particles when caring for patients who may have risks of viral transmission, but data are lacking (McGrath et al., 2020; Zaga et al., 2020). The benefits of PMV and other one-way valves for speech and reducing aspiration risk must be carefully weighed against the above risk, especially for speech-language pathologists who have significant exposure to mucosal surfaces and secretions. As tracheostomy is usually be followed by long periods of functional dependency and rehabilitation, the importance of multidisciplinary team-based approach should be valued and prospective data are needed to further guide best practices in tracheostomy care during the current pandemic.

### Limitations

Our study has several limitations. First, long-term follow-up on the effect of PMV intervention and the effect of PMV on aspiration pneumonia is lacking and still needs to be explored in future studies. Second, whether aspiration after tracheostomy in patients with ABI is due to changes in tracheostomy or brain damage caused by late neuronal dysfunction is uncertain, which also requires a no-aspiration group after tracheostomy in ABI for an in-depth, systematic comparative study. Third, although the proximal cause of tracheotomy status in the patient population was ABI, this diagnosis and indication does not exclude the possibility of other concurrent pathology. For instance, we have not performed other diagnostic procedures, such as laryngoscopy or bronchoscopy, to evaluate the structural anatomic factors that might have contributed to need for tracheostomy or increased aspiration risk. Lastly, other covariate factors related to aspiration were not controlled for, including smoking, hypertension, chronic obstructive pulmonary disease, gastroesophageal reflux disease, and psychological factors. Further research is required to control for these confounding factors.

## Conclusion

Our findings revealed that VP-Max, UES-RD, and aspiration in tracheostomized patients could be improved by PMV, and the putative mechanism may involve the subglottic pressure. In the setting of the COVID-19 pandemic, the benefits of PMV and other one-way valves for speech and reducing aspiration risk must be carefully weighed against the risk, such as aerosol generation in patients who may have risks of viral transmission. The importance of multidisciplinary team-based approach should be valued and prospective data are needed to further guide best practices in tracheostomy care.

## Data availability statement

The original contributions presented in this study are included in the article/supplementary material, further inquiries can be directed to the corresponding author/s.

## Ethics statement

The studies involving human participants were reviewed and approved by the Ethics Committee of The Third Affiliated Hospital of Sun Yat-sen University. The patients/participants provided their written informed consent to participate in this study.

## Author contributions

ZD: full access to all of the data in the study and takes responsibility for the integrity of the data, the accuracy of the data analysis, study supervision, and organization of the project. XH, QY, ZM, DP, and ZD: study concept and design. XH and QY: drafting of the manuscript. XW and HW: critical revision of the manuscript for important intellectual content. All authors contributed to the article and approved the submitted version.
